# Glycogen storage disease type Ia (GSDIa) but not Glycogen storage disease type Ib (GSDIb) is associated to an increased risk of metabolic syndrome: possible role of microsomal glucose 6-phosphate accumulation

**DOI:** 10.1186/s13023-015-0301-2

**Published:** 2015-07-29

**Authors:** Daniela Melis, Alessandro Rossi, Rosario Pivonello, Mariacarolina Salerno, Francesca Balivo, Simona Spadarella, Giovanna Muscogiuri, Roberto Della Casa, Pietro Formisano, Generoso Andria, Annamaria Colao, Giancarlo Parenti

**Affiliations:** Department of Pediatrics, Azienda Ospedaliera Universitaria “Federico II”, Via Sergio Pansini, 5 80131 Naples, Italy; Department of Translational Medical Sciences, Section of Pediatrics, “Federico II” University, Naples, Italy; Department of Medicine and Surgery, Section of Endocrinology, “Federico II” University, Naples, Italy; Department of Translational Medical Sciences, Section of Clinical Pathology, “Federico II” University, Naples, Italy

**Keywords:** GSDI, 11ΒHSD1, Metabolic syndrome, Insulin-resistance

## Abstract

**Background:**

In GSDIa, glucose 6-phosphate (G6P) accumulates in the endoplasmic reticulum (ER); in GSDIb, G6P levels are reduced in ER. G6P availability directly modulates the activity of 11β-hydroxysteroid dehydrogenase type 1 (11βHSD1), an ER-bound enzyme playing a key role in the development of the metabolic syndrome (MS).

**Objective:**

To evaluate the prevalence of MS and Insulin Resistance (IR) in GSDIa and GSDIb patients.

**Patients and Methods:**

This was a prospective study. All the enrolled patients were followed at the Department of Pediatrics “Federico II” University of Naples for 10 years. Clinical and biochemical parameters of MS and the presence of IR were recorded. The results were correlated with the biochemical parameters of GSDI-related metabolic control. 10 GSDIa patient (median age 12.10 ± 1.50), 7 GSDIb patients (median age 14.90 ± 2.20 were enrolled in the study. They were compared to 20 and 14 age and sex matched controls, respectively. 10 GSDIa patients (median age 24.60 ± 1.50) and 6 GSDIb patients (median age 25.10 ± 2.00) completed the 10-year-follow-up. At the end of the study the patients’ data were compared to 10 and 6 age and sex matched controls, respectively.

**Results:**

At study entry, 20 % GSDIa patients had MS and 80 % showed 2 criteria for MS. GSDIa patients showed higher HOMA-IR than controls and GSDIb patients (*p* < 0.001, *p* < 0.05), respectively. Baseline ISI was lower in GSDIa than controls (*p* < 0.001). QUICKI was significantly lower in GSDIa than in controls (*p* < 0.001). At the end of the study 70 % of GSDIa patients had MS and 30 % showed 2 criteria for MS. HOMA-IR was higher in GSDIa than controls (*p* < 0.01). Baseline ISI was higher in GSDIb than controls (*p* < 0.005) and GSD1a (*p* < 0.05). QUICKI was lower in GSD1a patients than in controls (*p* < 0.03). VAI was higher in GSDIa patients than controls (*p* < 0.001) and GSDIb patients (*p* = 0.002).

**Conclusions:**

Our data showed high prevalence of IR and MS in GSDIa patients. We speculate a possible role of 11βHSD1 modulation by G6P availability. We suggest a routine metabolic assessment in GSDIa patients.

## Background

Metabolic syndrome (MS), one of the most common clinical conditions nowadays, represents a combination of cardiometabolic risk determinants, including visceral obesity, insulin resistance (IR), hypertension, glucose intolerance or diabetes and dyslipidemia [1]. Recent studies in humans and rodents suggest a role of 11β-hydroxysteroid dehydrogenase (11β-HSD) in the development of idiopathic obesity and MS [2]. The increased 11β-HSD1 activity in adipose tissue in obese rats and in some but not all studies of obese humans causes visceral obesity and its metabolic consequences [3].

Mouse model with selective liver 11β-HSD1 overexpression show IR, dyslipidemia, and hypertension, but unaltered adiposity. The elevated levels of insulin detected in response to glucose challenge together with increased fasting insulin levels in older mice suggest that they progressively develop IR. The mechanisms involve both direct effects on target gene expression in the insulin signaling pathway and the alteration of other key transcriptional regulators of lipid homeostasis [4].

Conversely, 11β-HSD1 null mice exhibit a protective glycemic, lipid, and lipoprotein profile and show increased expression of hepatic mRNAs encoding regulators of fatty acid beta-oxidation. These 11βHSD1 knock-out mice are resistant to the development of MS [5–7]. In addition pharmacological inhibition of 11βHSD1 has been associated to beneficial effects on weight, glycemic control and lipid profile in humans [8, 9].

11βHSD1 is an ER-bound enzyme catalyzing the conversion of inactive cortisone in active cortisol in humans. It is typically expressed in glucocorticoid receptors-rich tissues, such as the liver, adipose tissue, lung and brain. 11βHSD1 requires NADPH as a cofactor generated by the hexose-6-phosphate dehydrogenase (H6PDH)-mediated conversion of glucose 6-phosphate (G6P) to 6-phosphogluconactone (6PGL) [10]. Down-regulation of hepatic 11βHSD1 transcription has been observed in diabetic mice transfected with glucose-6-phosphate translocase (G6PT) antisense oligonucleotides [11].

Glucose-6-phosphatase (G6Pase) system catalyzes the hydrolysis of glucose 6-phosphate (G6P) to glucose and inorganic phosphate. It is a multicomponent system of proteins located in the endoplasmic reticulum that comprises a catalytic subunit (G6PC) and the transporter for G6P (G6PT). G6PT (encoded by *SLC37A4* gene) translocates G6P, the product of gluconeogenesis and glycogenolysis, from the cytoplasm to the lumen of the endoplasmic reticulum (ER), where G6P is converted into glucose and phosphate by G6PC [12, 13].

The *G6PC* gene is expressed predominantly in neoglucogenetic organs such as liver, kidney, lower levels in intestine and also pancreatic islets. Mutations of *G6PC* causes glycogen storage disease type 1a (GSD1a, MIM23.2200), whereas mutations of *SLC37A4* causes GSD type 1b (GSD1b, MIM23.2200). Since the G6Pase complex has a key role in glycogenolysis and gluconeogenesis, both disorders are characterized by a typical metabolic profile with fasting hypoglycemia, hepatomegaly, nephromegaly, hyperlacticacidemia, hyperlipidemia, overweight and hyperuricemia [14]. Long term complications include: hepatic adenomas, renal failure and neurocognitive dysfunction [15]. In addition, GSD1b patients present neutropenia and/or neutrophil dysfunction, great susceptibility to recurrent bacterial infections and an increased risk of autoimmune disease [16].

It has been previously shown that G6P availability directly modulates 11βHSD1 activity. In GSDIa, the G6P excess in ER (due to G6Pase deficiency) has been associated to increased 11βHSD1 activity, while in GSDIb the lack of G6P in ER (due to G6PT deficiency) has been associated to decreased 11βHSD1 activity [17].

Therefore it may be hypothesized that GSD1a patients are at risk to develop MS, conversely GSD1b should be protected from this complication.

The aim of the present study was to evaluate the prevalence of MS and IR in GSDIa and GSDIb patients.

### Patients

Seventeen GSDI patients were enrolled. They represent the entire case load of GSD1 patients followed at the Department of Pediatrics “Federico II” University of Naples. No selection criteria were considered. At study entry GSDIa patients (4 males and 6 females) had a median age of 12.10 ± 1.50. GSDIb patients (2 males and 5 females) had a median age of 14.90 ± 2.20. These patients were followed for a 10 year-period. At the end of the study GSD1a patients (4 males and 6 females) had a median age of 24.60 ± 1.50 and GSD1b patients (2 males and 4 females) a median age of 25.10 ± 2.00. One GSD1b patient died during follow-up. The diagnosis of GSDIa and GSDIb was based on mutation analysis of the G6PC and SLC37A4 gene, respectively. All patients were on dietary treatment.

### Study design

The study protocol was in accordance with the Italian regulations on privacy protection and with the Helsinki Doctrine for Human Experimentation.

Seventeen GSDI patients were enrolled. At study entry, GSDIa patients (4 males and 6 females, median age 12.10 ± 1.50) were compared to 20 age and sex matched controls. GSDIb patients (2 males and 5 females, median age 14.90 ± 2.20) were compared to 14 age and sex matched controls. The patients were followed for a 10 year-period. Biochemical data were recorded at study entry, every year during follow-up and at the end of the study. At the end of the study GSD1a patients (4 males and 6 females, median age 24.60 ± 1.50) and GSD1b patients (2 males and 4 females median age 25.10 ± 2.00) were compared to 16 controls (6 males and 10 females median age 26.10 ± 1.70).

To investigate the prevalence of MS in GSD1 patients, MS criteria in according to International Diabetes Federation (IDF) guidelines were recorded. IR is a hallmark of obesity and MS. Therefore, quantitative assessment of IR is of a great importance for detecting its presence and assessing its severity. Various methods are currently employed. Direct methods (e.g. hyperinsulinemic euglycemic glucose clamp) are the reference techniques. However they are complex, time-consuming and invasive procedures. Surrogate indexes are the most commonly used. They represent inexpensive quantitative tools that can be easily applied in clinical research investigations and clinical practice. To minimize the limitations of each index more than one index should be used simultaneously [18]. Among these Homeostasis model assessment of Insulin Resistance (HOMA-IR), Insulin sensitivity index (ISI) and Quantitative insulin sensitivity check index (QUICKI) have been extensively validated [19, 20]. Recently the visceral adiposity index (VAI) has been proposed as an indicator of IR and adipose tissue dysfunction. VAI is a sex-specific mathematical index based on Waist circumference (WC), Body Mass Index (BMI), triglycerides (TG) and HDL cholesterol (HDL-C) that showed a strong association with both the rate of peripheral glucose utilization and visceral adipose tissue [21]. VAI has been proposed as an easy tool for early detection of a condition of cardiometabolic risk. One important limitation to consider is the application in patient aged less than 16 years: VAI should not be applied in this age range because the numerical factors considered are derived from healthy adult population [21].

In the present study IR was assessed by evaluation of HOMA-IR, baseline ISI, QUICKI and VAI.

At study entry, considering that the majority of patients were children they had shorter fasting time than controls. In addition considering that the majority of patients were aged less than 16 years, VAI was evaluated only in 3 GSDIa and in 3 GSDIb patients. To overcome the bias due to patients short fasting time, at the end of the study, when all the patients were adult, the control subjects were asked to have blood sampling after the same fasting time of his/her age and sex matched patient.

## Methods

### Clinical and biochemical parameters of GSD1-related metabolic control

The following clinical parameters were recorded: height SDS score, BMI, waist circumference WC, systolic and diastolic blood pressure (BP). Biochemical parameters included: fasting plasma glucose (FPG), TG, cholesterol, lactic and uric acid levels, bicarbonate, baseline fasting insulin serum levels. The compliance to the dietary or medical treatment was also recorded.

### Metabolic syndrome and IR assessment

MS was defined according to IDF guidelines. For children and adolescents (age less than 16 years) to be defined as having the MS the patients must have at least 3 of the following criteria: WC > 90^th^ percentile, TG > 150 mg/dL, HDL-C < 40 mg/dL, systolic BP > 130 mmHg or diastolic BP > 85 mmHg, fasting plasma glucose (FPG) > 100 mg/dL or known type 2 diabetes mellitus. For adults (age more than 16 years) to be defined as having the MS the patients must have at least 3 of the following criteria: WC > 94 cm in males and >80 cm in females, TG > 150 mg/dL, HDL-C < 40 mg/dL in males and < 50 mg/dL in females, systolic BP > 130 mmHg or diastolic BP > 85 mmHg, FPG: > 100 mg/dL or previously diagnosed type 2 diabetes.

HOMA-IR was calculated as following: (FPG mmol/L x Fasting Insulin μU/mL)/ 22.5. Basing on HOMA-IR values patients were divided into two groups: non-IR (HOMA-IR < 2.5) and IR (HOMA-IR > 2.5) [22].

Baseline ISI was calculated as following = 10,000/(fasting insulin [μU/ml] × FPG [mg/dl]). QUICKI was calculated as following: 1/[Log Fasting Insulin μU/mL + Log FPG mg/dL].

VAI was calculated as following: males VAI = {WC cm/[39.68 + (1.88 × BMI)]} x (TG mmol/L/1.03) x (1.31/HDL mmol/L), females VAI = {WC cm/[36.58 + (1.89 × BMI)]} x (TG mmol/L/0.81) x (1.52/HDL mmol/L). Cut-off was set at 2.52 [23].

### Statistical analysis

All data in the text or shown in the figures are expressed as mean ± SE. Statistical analysis was performed using Statistical Package for Social Science (SPSS 10 for Windows Update; SPSS Inc., Chicago, Illinois, USA). The comparisons between numerical variables were performed by Student’s t-test corrected for Fisher’s exact test. Correlation study was performed by Pearson test. Statistical significance was set at *p* < 0.05.

## Results

### Clinical and biochemical parameters of GSD1-related metabolic control

At study entry, height SDS was lower in both GSDIa (−1.00 ± 0.30 vs −0.20 ± 0.14, *p* < 0.05) and GSDIb patients (−1.27 ± 0.50 vs 0.58 ± 0.20, *p* < 0.001) than in controls. No significant difference was observed in the other clinical parameters between GSDIa and GSDIb patients and controls. In GSDIa patients serum cholesterol, TG, lactic acid, uric acid and insulin levels were higher than in controls. In GSDIb serum cholesterol levels were significantly lower, lactic acid and uric acid higher than in controls (Table 1).

**Table 1:** Biochemical parameters of metabolic control in GSDI patients and controls at study entry

	GSDIa		Controls		GSDIb		Controls		Significance (p)		
	Mean	SE	Mean	SE	Mean	SE	Mean	SE	Ia vs C	Ib vs C	Ia vs Ib
Glucose (mg/dL)	92.80	17.79	85.70	5.92	106.71	27.43	91.57	10.58	0.11	0.08	0.22
Cholesterol (mg/dL)	191.40	35.30	149.00	23.00	104.60	15.02	139.00	32.30	0.001	0.02	0.001
Triglycerides (mg/dL)	379.10	176.10	88.20	35.30	116.40	74.69	108.00	38.30	0.001	0.73	0.001
Lactic acid (mg/dL)	2.16	0.47	1.33	0.22	3.26	1.78	1.35	0.22	0.001	0.001	0.08
Uric acid (mg/dL)	5.10	0.94	3.82	1.25	6.17	1.49	3.79	0.88	0.01	0.001	0.09
Bicarbonate (mEq/L)	22.40	2.24	26.31	1.91	24.60	13.00	24.57	1.79	0.001	0.98	0.60
Insulin (μU/L)	29.70	21.60	8.47	3.69	14.69	13.11	8.46	2.03	0.001	0.09	0.12

At the end of the study GSDIa patients showed higher BMI (25.94 ± 1.10 vs 22.49 ± 1.06, *p* < 0.05) and WC (95.08 ± 2.99 vs 77.90 ± 0.75, *p* < 0.001) than controls. In GSDIa patients serum cholesterol, TG, lactic acid, uric acid and insulin serum levels were higher than in controls. In GSD1b patients, lactic acid and uric acid were significantly higher than in controls, serum TG and cholesterol were lower than GSD1a (Table 2).

**Table 2:** Biochemical parameters of metabolic control in GSDI patients and controls at the end of the study

	GSDIa		GSDIb		Controls		Significance (p)		
	Mean	SE	Mean	SE	Mean	SE	Ia vs C	Ib vs C	Ia vs Ib
Glucose (mg/dL)	84.43	4.50	88.60	8.00	90.37	1.02	0.22	0.46	0.65
Cholesterol (mg/dL)	253.89	7.76	130.2	16.43	139.75	4.38	0.001	0.45	0.006
Triglycerides (mg/dL)	481.20	39.19	135.00	53.90	99.40	8.52	0.001	0.16	0.01
Lactic acid (mg/dL)	5.11	0.38	4.52	1.22	1.33	0.05	0.001	0.001	0.78
Uric acid (mg/dL)	4.79	0.12	6.10	1.10	3.54	0.24	0.007	0.001	0.18
Bicarbonate (mEq/L)	22.14	0.17	23.74	1.41	24.22	0.48	0.01	0.67	0.23
Insulin (μU/L)	19.40	2.67	9.28	0.58	5.02	0.39	0.04	0.14	0.47

### Metabolic syndrome and Insulin Resistance assessment

**Fig. 1:**
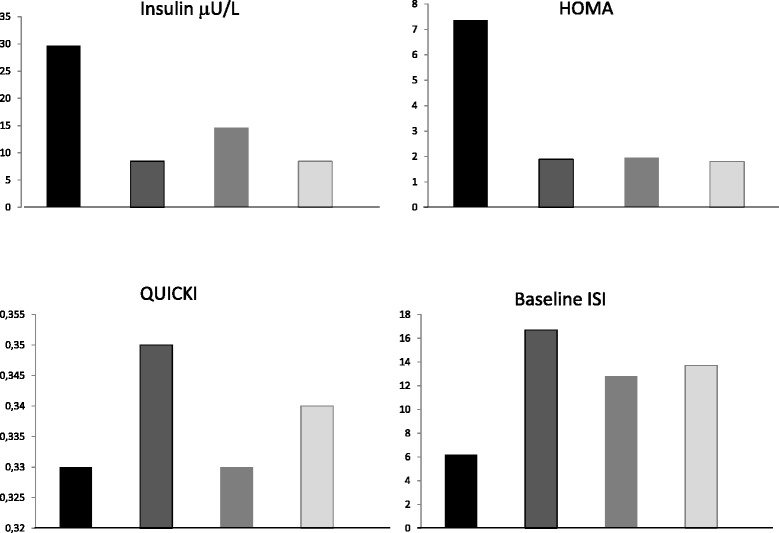
Mean value of insulin serum levels, HOMA-IR, QUICKI and baseline ISI levels in GSDIa patients (Black rectangle), GSDIa-matched controls (Black/gray rectangle), GSDIb patients (Gray) and GSDIb-matched controls (dark gray/gray rectangle)

**Fig. 2:**
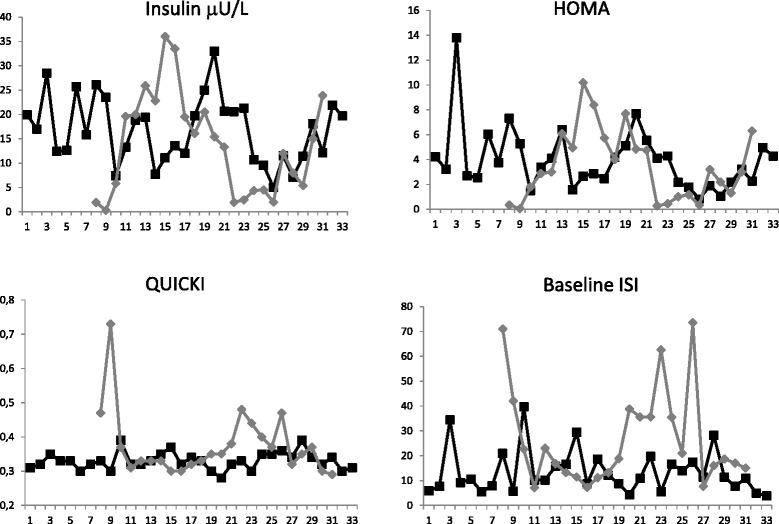
Mean value of insulin serum levels, HOMA-IR, QUICKI and baseline ISI levels in GSDIa patients (Black square) and GSDIb patients (Gray diamond) during the 10-year follow-up

At study entry 2/10 (20 %) GSDIa and 0/7 (0 %) GSDIb patients had MS. The remaining 8/10 (80 %) GSDIa patients showed 2 criteria for MS. Among GSDIb patients, 3/7 (43 %) showed 2 criteria, 1/7 (14 %) showed 1 criterion, 3/7 (43 %) did not show any criteria. Considering HOMA-IR, among GSDIa patients, 8/10 (80 %) were IR and 2/10 (20 %) were non-IR; in GSDIb patients 2/7 (29 %) were IR and 5/7 (71 %) were non-IR. HOMA-IR values were higher in GSDIa patients than controls (7.36 ± 2.13 vs 1.90 ± 0.13, *p* < 0.001) and GSDIb patients (7.36 ± 2.13 vs 1.97 ± 0.40, *p* < 0.05). No significant difference in HOMA-IR was observed between GSDIb patients and controls. Baseline ISI values were significantly lower in GSDIa than in controls (6.21 ± 0.44 vs 16.76 ± 1.85, *p* < 0.001). No significant difference was detected between GSDIb patients and controls (12.86 ± 4.41 vs 13.74 ± 0.92, *p* = 0.79). QUICKI value was significantly lower in GSDIa than in controls (0.30 ± 0.003 vs 0.36 ± 0.006, *p* < 0.001) (Fig. 1). No significant difference was detected between GSDIb patients and controls (0.33 ± 0.01 vs 0.35 ± 0.003, *p* = 0.20). VAI data were available only for 3 GSDIa patients (in 2/3 VAI was >2.52) and 3 GSDIb (in 2/3 VAI was >2.52). During the 10-year follow-up 6/10 GSD1a (5 female, 1 male) patients showed increased serum insulin levels in particular during puberty and adulthood. Conversely only 2/7 GSDIb showed increased insulin serum levels (1 male, 1 female). Fig. 2 shows mean value of serum insulin levels, HOMA-IR, ISI and QUICKI during the follow-up. The Fig. 2 shows increased insulin serum levels and HOMA-IR especially in GSDIa. Conversely in GSDIb patients, high QUICKI and ISI were recorded during follow-up.

At the end of the study all the enrolled patients were adult. Among them, 7/10 (70 %) GSDIa and 0/7 (0 %) GSDIb patients had MS. The remaining 3/10 (30 %) GSDIa patients showed 2 criteria for MS. Among GSDIb patients, 2/6 (33 %) showed 2 criteria, 4/6 (66 %) showed 1 criterion.

Considering HOMA-IR in GSDIa patients, 6/10 (60 %) were IR and 4/10 (40 %) were non-IR; in GSDIb patients 2/6 (33 %) were IR and 4/6 (66 %) were non-IR. HOMA-IR value was higher in GSD1a patients than in controls (4.75 ± 1.80 vs 1.11 ± 0.10, *p* < 0.01). No significant difference in HOMA-IR was observed between GSDIb patients and controls.

Baseline ISI values were significantly higher in GSDIb than in controls (54.90 ± 7.00 vs 24.33 ± 1.97, *p* < 0.005) and GSDIa (18.41 ± 0.37, *p* < 0.05).

**Fig. 3:**
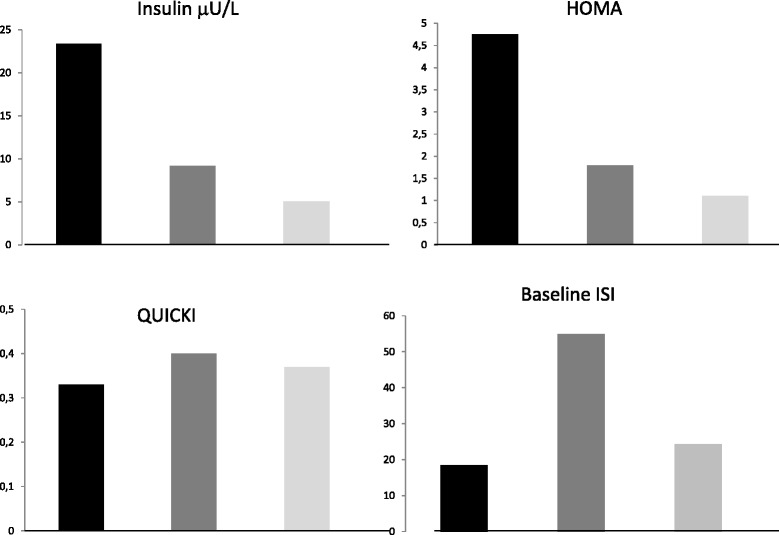
Mean value of insulin serum levels, HOMA-IR, QUICKI and baseline ISI levels in GSDIa patients (Black rectangle), GSDIb patients (Gray rectangle) and controls (Light gray rectangle) at the end of the study

QUICKI values were lower in GSDIa patients than in controls (0.33 ± 0.008 vs 0.37 ± 0.005, *p* < 0.05) (Fig. 3).

VAI value was significantly higher in both GSDIa (8.67 ± 0.48, vs 1.63 ± 0.16, *p* < 0.001) and GSDIb patients (3.76 ± 1.12 vs 1.63 ± 0.16, *p* < 0.005) than in controls.

#### Correlation study

In GSD1a patients HOMA data inversely correlated with both QUICKI (*r* = −0.69, *p* < 0.001) and ISI results (*r* = −0.45, *p* < 0.05); QUICKI also correlated with ISI data (*r* = 0.94, *p* < 0.0001).

In GSD1b patients HOMA data inversely correlated with both QUICKI (*r* = −0.76, *p* < 0.005) and ISI results (*r* = −0.68, *p* < 0.01); QUICKI also correlated with ISI data (*r* = 0.97, *p* < 0.0001).

## Discussion

GSDI is a rare and genetically heterogeneous inborn error of metabolism. Two forms of the disease have been identified: GSDIa, caused by mutations of the G6PC gene, encoding glucose-6-phosphatase expressed in liver, kidney and bowel, and GSDIb, caused by mutations of the SLC37A4 gene, encoding glucose-6-phosphate translocase ubiquitously expressed.

Both genetic defects result in the block of the final steps of gluconeogenesis and glycogenolysis, reducing the release of glucose from glycogen in response to fasting, and causing glycogen accumulation in the liver and in the kidney. The clinical manifestations of the metabolic derangement are hepato/nephromegaly and reduced tolerance to fasting, associated with hypoglycemia, lactic acidosis, hypertriglyceridemia, hyperuricemia. In addition to the clinical features of GSDIa, most GSDIb patients have neutropenia and neutrophil dysfunction that predispose to severe infections and to inflammatory bowel disease (IBD).

The treatment of GSDI is aimed at maintaining normoglycemia and consists of dietary regimens based on frequent meals, cornstarch supplementation and/or nocturnal gastric drip feeding [24]. Diet is highly effective in correcting the metabolic derangement of the disease.

With the use of dietary treatment, life expectancy improved. However, a number of systemic complications, such as hepatic adenomas, renal failure and osteoporosis impact heavily on patients’ prognosis and quality of life [25].

G6Pase system catalyzes the hydrolysis of G6P to glucose and inorganic phosphate [12]. It has been previously shown that G6P availability directly modulates 11βHSD1 activity [17]. The accumulation of G6P in ER fuels the G6PT-H6PDH-11βHSD1 system, leading to increased prereceptorial activation of glucocorticoids [26, 27]. The effect of glucocorticoids on insulin sensitivity and their role in the pathophysiology of IR and MS are clearly known [28], suggesting a possible link between 11βHSD1 activity, IR and MS.

We speculated that in GSDIa patients, G6P excess in liver ER (due to G6Pase deficiency) would increase 11βHSD1 activity causing IR and MS. Conversely in GSDIb patients, with low ER G6P levels that would reduce 11βHSD1 activity, an increased insulin sensitivity was expected.

In the present study we analyzed the presence of MS criteria, visceral adiposity, and IR in 10 GSDIa and 7 GSDIb patients. We found that GSDIa patients showed an increased prevalence of MS. Indeed it cannot be excluded that obesity as well as hyper-triglyceridemia detected in GSDIa are due to the inborn error of metabolism. Moreover one of the criterion for MS is hyperglycemia that would be never present in GSDI patients. Probably these criterion for the diagnosis of MS cannot be used for GSDI patients.

Indeed, since G6Pase, mutated in GSDIa patients is expressed only in liver, bowel and kidney, we hypothesized the presence of IR without obesity as is the case of mouse model with selective liver 11β-HSD1 overexpression, showing insulin resistance, dyslipidemia, and hypertension, but unaltered adiposity. These mice show fat accumulation in the liver, mainly as TG. The association of IR and fatty liver irrespective of obesity has been proposed as an early indicator of primary hepatic IR preceding more widespread IR and the full MS. Both lipogenesis and lipid oxidation are activated in these mice with an apparent net accumulation of lipid in liver and serum (4).

In GSDIa patients we demonstrated the presence of high serum insulin levels, high HOMA-IR, as well as low QUICKI and baseline ISI both at the first evaluation or during the entire 10-year follow-up. To overcome the bias due to patients’ short fasting time, at the end of the study, the adult control subjects were asked to have blood sampling after the same fasting time of his/her age and sex matched patient. Also, at the end of the study GSDIa patients showed higher serum insulin levels and HOMA-IR and lower QUICKI and ISI than controls demonstrating IR. Indeed GSD1a patients also showed serum TG significantly higher than both controls and GSD1b. This data might confirm a role of the modulation of 11β-HSD1 activity by G6P in GSD1a patients.

Several studies showed that inhibition of 11βHSD1 reduces glucose intolerance, IR and plasma TG levels in preclinical models of MS [6, 7]. On the basis of these data we hypothesized that GSD1b patients are “protected” from IR and MS.

Indeed GSDIb patients did not show a high prevalence of MS; at the end of the study no patient showed MS. At study entry no significant difference in HOMA-IR, baseline ISI and QUICKI was observed between GSDIb patients and controls. During the 10-year follow-up 6/10 GSDIa patients showed increased serum insulin, conversely only 2/7 GSDIb showed increased insulin serum levels. In adult GSDIb patients, baseline ISI was significantly higher than in both controls and GSDIa suggesting low IR. In addition, pharmacological inhibition of 11βHSD1 has been associated to beneficial effects on weight, glycemic control and lipid profile in humans [8, 9]. The interference with 11β HSD1 activity might also explain the absence, in GSD1b patients, of hypertriglyceridemia which is part of the biochemical features of the inborn error of metabolism, namely GSD.

VAI data resulted significantly higher in both groups of patients than in controls. The presence of obesity and high serum TG levels is part of the clinical and biochemical features of the GSDI. A role of the dietary treatment, including frequent meal, continuous enteral feeding and/or corn starch cannot be excluded. However it is noteworthy that in GSDIb patients, BMI was similar to control subject and that hypertriglyceridemia was exclusively present in GSDIa patients.

The results of the present study showing increased IR in GSDIa patients and increased insulin sensitivity in GSDIb patients suggest a role of the interaction between G6P and 11βHSD1.

## Conclusion

In conclusion, we demonstrated that GSDIa patients have IR and are at risk for developing MS. These results shed new light on so far unrecognized complications, such as IR and MS and have obvious clinical implications, suggesting a routine metabolic assessment in the management of GSDIa patients.

In addition, we emphasize the differences between GSDIa and GSDIb, contributing once more to consider and to treat them as different entities.

## Abbreviations

G6P: Glucose 6-phosphate
11βHSD1: 11β-hydroxysteroid dehydrogenase
MS: Metabolic syndrome
IR: Insulin-resistance
HOMA-IR: Homeostasis model assessment of Insulin Resistance
ISI: Insulin sensitivity index
QUICKI: Quantitative insulin sensitivity check index
VAI: Visceral adiposity index
